# Predictive Models for Suicide Attempts in Major Depressive Disorder and the Contribution of *EPHX2*: A Pilot Integrative Machine Learning Study

**DOI:** 10.1155/2024/5538257

**Published:** 2024-05-09

**Authors:** Shuqiong Zheng, Weixiong Zeng, Qianyun Wu, Weimin Li, Zilong He, Enze Li, Chong Tang, Xiang Xue, Genggeng Qin, Bin Zhang, Honglei Yin

**Affiliations:** ^1^Department of Psychiatry, Sleep Medicine Center, Nanfang Hospital, Southern Medical University, Guangzhou, China; ^2^Institute of Brain Disease, Nanfang Hospital, Southern Medical University, Guangzhou, China; ^3^Key Laboratory of Mental Health of the Ministry of Education, Southern Medical University, Guangzhou, China; ^4^Department of Radiology, Nanfang Hospital, Southern Medical University, Guangzhou, China; ^5^Department of Psychiatry, The Third Affiliated Hospital, Southern Medical University, Guangzhou, China

## Abstract

Suicide is a major public health problem caused by a complex interaction of various factors. Major depressive disorder (MDD) is the most prevalent psychiatric disorder associated with suicide; therefore, it is essential to prioritize suicide prediction and prevention within this population. Integrated information from different dimensions, including personality, cognitive function, and social and genetic factors, is necessary to improve the performance of predictive models. Besides, recent studies have indicated the critical roles for EPHX2/P2X2 in the pathophysiology of MDD. Our previous studies found an association of *EPHX2* and *P2X2* with suicide in MDD. This study is aimed at (1) establishing predictive models with integrated information to distinguish MDD from healthy volunteers, (2) estimating the suicide risk of MDD, and (3) determining the contribution of *EPHX2*/*P2X2*. This cross-sectional study was conducted on 472 prospectively collected participants. The machine learning (ML) technique using Extreme Gradient Boosting (XGBoost) classifier was employed to evaluate the performance and relative importance of the extracted characteristics in recognising patients with MDD and depressed suicide attempters (DSA). In independent validation set, the model with clinical and cognitive information could recognise MDD with an area under the receiver operating characteristic curve (AUC) of 0.938 (95% confidence interval (CI), 0.898–0.977), and genetic information did not improve classification performance. The model with clinical, cognitive, and genetic information resulted in a significantly higher AUC of 0.801 (95% CI, 0.719–0.884) for identifying DSA than the model with only clinical information, in which the three single nucleotide polymorphisms of *EPHX2* showed important roles. This study successfully established step-by-step predictive ML models to estimate the risk of suicide attempts in MDD. We found that *EPHX2* can help improve the performance of suicidal predictive models. This trial is registered with NCT05575713.

## 1. Introduction

Suicide, a self-destructive behavior with the exact intent to die, is a worldwide public health problem [1] Suicide leads to more than 800,000 people die each year, as estimated by the World Health Organization [2]. Accurate prediction for suicide attempts requires the assessment for the interactions of various risk factors [3]. The primary challenge in accurately predicting suicide attempts stems from their low occurrence rate. Concentrating on high-risk subgroups, such as individuals with major depressive disorder (MDD), could offer a solution to this issue [4, 5]. Notably, MDD is a significant contributor to the disease burden, characterized by an exceptionally high risk of suicide [6]. Early recognition of depressive symptoms is one of the critical facets in suicide prevention and may save the lives of patients with MDD [6, 7]. Therefore, focusing on subgroups of patients with MDD to establish a suicidal predictive model can improve our ability in suicide prediction and prevention.

As conventional interview-based diagnoses are insufficient for accurately predicting suicide attempts, machine learning (ML) has been widely used in suicide research to produce clinically useful models of future suicide in recent studies [8]. The limitation of most of these studies was the single subject area, such as demographic, interpersonal, and social factors, functional magnetic resonance imaging, or electroencephalography [3, 9–12]. Furthermore, our previous study found that patients with suicide behavior performed more conservatively in decision-making under ambiguous conditions compared to the HC group and established a clinically useful predictive model with the information of cognitive function for predicting suicide attempts among MDD patients by ML [13]. However, the significant causes of MDD and suicide attempts involve the complex interaction of multiple variables, including personality, cognitive function, and social and genetic factors [12]. Thus, integrated information from different dimensions is necessary to improve the performance of predictive models.

A series of large cohort studies and genome-wide association study (GWAS) had provided convincing evidence for the heritability of MDD and suicide but have not produced consistent results [14–16]. A GWAS in the UK Biobank identified significant single nucleotide polymorphism- (SNP-) based heritability in patient-reported suicide attempt phenotypes using ML [17]. Another study found that gene expression can be used to evaluate depression and suicide risk [18]. Recent studies have indicated the critical roles for *EPHX2*/*P2X2* in the pathophysiology of depression [19–22]. Our previous study revealed the association between *EPHX2*/*P2X*2 and suicide attempts [23]. However, no study has determined whether SNP or gene expression of *EPHX2*/*P2X2* can help predict and prevent suicide using ML.

Therefore, this study aimed at establishing a predictive model with integrated information, including genetic, environmental, cognitive, and psychometric properties, to (1) distinguish patients with MDD from healthy controls, (2) estimate the suicide risk of MDD, and (3) determine whether *EPHX2*/*P2X2* could improve the performance of predictive models.

## 2. Materials and Methods

### 2.1. Study Design and Participants

**Figure 1:**
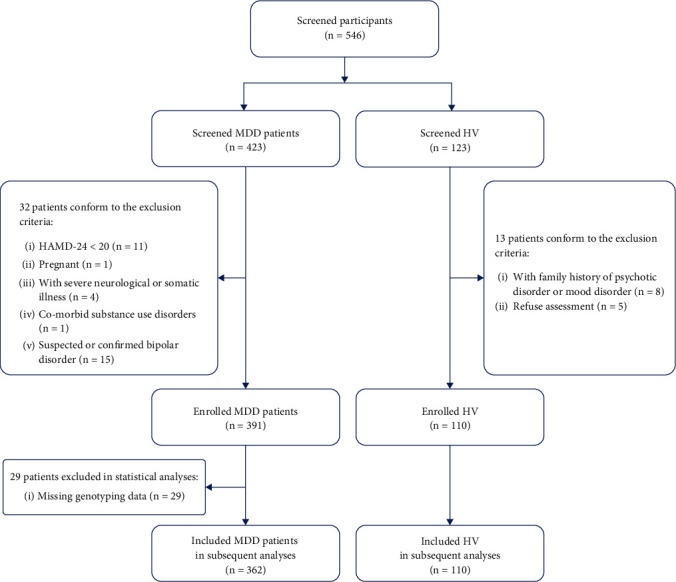
The inclusion and exclusion criteria of the study.

This cross-sectional study used the same cohorts of patients and tissue samples as those in our previous study [23]. This study adhered to the transparent reporting of a multivariable prediction model for individual prognosis or diagnosis (TRIPOD) guidelines. The inclusion and exclusion criteria are described in Figure 1. The data were collected between June 2019 and September 2021. In total, 501 participants, including 391 MDD patients and 110 healthy volunteers (HV), were screened. The HV were recruited through advertisements.

Two experienced clinical psychiatrists interviewed outpatients at the Department of Psychiatry at Nanfang Hospital (Guangzhou, China). The agreement between the two psychiatrists was estimated using Cohen kappa coefficients.

At baseline, MDD were diagnosed by the fifth edition of the Diagnostic and Statistical Manual of Mental Disorders, and the severity of MDD was evaluated by the Hamilton MDD Scale-24 (HAMD-24). Patients with a score of >20 on HAMD-24 were enrolled in this study [24]. Additionally, patients using drugs including mood stabilizers, antidepressants, anxiolytics, antipsychotics, and benzodiazepines within the previous two weeks were excluded. Participants with a history of psychotic disorders, mood disorders, and suicidal intent or attempts were excluded from the HV group.

During the 12-month clinical follow-up period, patients with suspected hypomanic symptoms or confirmed bipolar disorder were excluded. Additionally, 29 patients were excluded owing to missing genotyping data. Finally, 362 patients with MDD (133 with depression and suicide attempt (DSA) and 229 with depression without suicide attempt (DNS)) and 110 HV were included in subsequent analyses.

### 2.2. Ethics Statements

This study was approved by the Ethics Review Committee of the Southern Hospital of Southern Medical University (approval number: NFEC-2022-092). All participants provided written consent before participating in the study.

### 2.3. Data Collection

#### 2.3.1. Clinic Interviews

Demographic and clinical information were collected. The severity of depressive symptoms was measured by the HAMD-24. The risk of suicide was measured by the Columbia-Suicide Severity Rating Scale [25]. Childhood trauma was measured by the Childhood Trauma Questionnaire (CTQ), which includes five subscales: emotional abuse, physical abuse, sexual abuse, physical neglect, and emotional neglect [26]. Impulsivity was measured by the Barratt Impulsiveness Scale (BIS-10), which includes three subscales: cognitive impulsiveness, motor, and nonplanning impulsiveness [27]. Aggressiveness was measured by the Buss-Perry Aggression Questionnaire (BPAQ), which includes four subscales: physical aggressiveness, verbal aggressiveness, anger, and hostility [28].

### 2.4. State-Dependent Test

#### 2.4.1. Cognitive Function

Three tasks were used to assess different domains of cognitive function. The Attention Network Test (ANT) was used to assess three dimensions of attention networks: alerting, orienting, and executive control [29]. The Suicide Stroop Task (SST) is used to assess executive function, processing speed, and attention bias [2]. Two versions of the n-back task (1-back and 2-back) were used to assess working memory [30]. The response time (RT) and accuracy (ACC) for each task were recorded. The stimulus presentation and data recording for the task were used by E-Prime 2.0 Professional SP1 (2.0.10.356) software.

### 2.5. Messenger RNA Expression Levels

The methodology for real-time polymerase chain reaction amplification and the associated calculations for *EPHX2* (gene ID: 2053) and *P2X2* (gene ID: 22953) have been delineated in our preceding study [23]. A previous study found that gene expression can more effectively represent emotional states, and a cognitive assessment was performed together with blood sample collection [18]. Thus, we regarded the relative expression levels of *EPHX2* and *P2X2* as information obtained from the state-dependent tests.

### 2.6. SNP Genotyping

The process of SNP selection and genotyping has been described in our previous study [23]. In our previous study, we identified ten SNPs within *EPHX2* and *P2X2* that showed associations with MDD and suicide attempts. For detailed information, refer to the Supplementary Methods (available here).

### 2.7. Data Preprocessing

This study established two datasets for the classification of patients with MDD vs. HV and those with DSA vs. DNS. Two datasets were constructed based on these three characteristics. For the classification tasks of MDD vs. HV, the first feature set included baseline demographic characteristics (sex, age, body mass index, marital status, education, family history of psychiatric disorder, MDD disorder, suicide, and history of psychiatric medicine use) and clinical questionnaires (CTQ, BIS, and BPAQ). The second feature set included cognitive function (ANT, 1-back, 2-back, and SST), and the RNA expression levels of *EPHX2* and *P2X2* were related to cognitive function. The third feature set encompassed the SNPs associated with MDD and suicide attempts, as identified in our prior research [23]. To better simulate the clinical situation, clinical information directly related to MDD was excluded from the first set of characteristics for the classification tasks of MDD vs. HV. For the classification tasks of DSA vs. DNS, the difference in the datasets was that some characteristics (recurrent major depressive episode, duration of current episode, age at onset, age at current episode, and HAMD-24 score) were added to the first set of characteristics.

The random forest interpolation [31] was used to supplement the missing values in the datasets. Its principle involves utilizing the other features within the same dataset to construct a random forest model. This model used the real values in the feature column containing missing values as the predictive target for training and subsequently conducts predictions for the missing values after multiple iterative processes. The proportion of missing values in the data was less than 20%. The detail of missing values can be found in the Supplementary Materials. The datasets were randomly divided into training and validation sets at a ratio of 7 : 3 using random stratified sampling. To enhance and expedite the training of the models, we performed data normalisation, which scales quantitative data to a range of 0–1. This normalisation process offers several advantages. Specifically, it facilitates faster model convergence and improves the overall performance of the models by addressing the issue of varying indicator levels [32, 33]. Finally, to reduce the data dimensions and to make the model more convenient and easier to use, we used an embedded method to select the features included in the training sets. This method enables ML algorithms to autonomously identify relevant characteristics, selecting features based on descending weight coefficients, and to construct various feature subsets for training and evaluation purposes. The outcomes of the embedded approach significantly enhanced the model's utility, thereby increasing its effectiveness.

### 2.8. ML Model Construction

This study used the Extreme Gradient Boosting (XGBoost) algorithm to complete the classification tasks established by the XGBoost library in Python 3.7. XGBoost is a highly effective and widely used ML algorithm in data science that achieves state-of-the-art results for many ML challenges [34]. It is an ensemble learning algorithm based on decision trees, excelling particularly in handling large datasets and featuring regularization to prevent overfitting effectively. During the training of models, we used tenfold cross-validation. A grid search algorithm was used to automatically traverse the prequalified parameter set and select the optimal hyperparameters using the scikit-learn library in Python. The ML workflow is shown in the Supplementary Materials.

To evaluate the added value of these characteristics, we established two sets of ML models with different input characteristics. Specifically, the models were based on the following input characteristics: (1) model A, information from clinical interviews (the first feature set); (2) model B, information from clinical interviews and state-dependent tests (the first and second feature set); and (3) model C, information from clinical interviews, state-dependent tests, and SNP genotyping (the first, second, and third feature set). After training was completed, we validated the classification performance of these machine learning models in independent validation sets.

**Figure 2:**
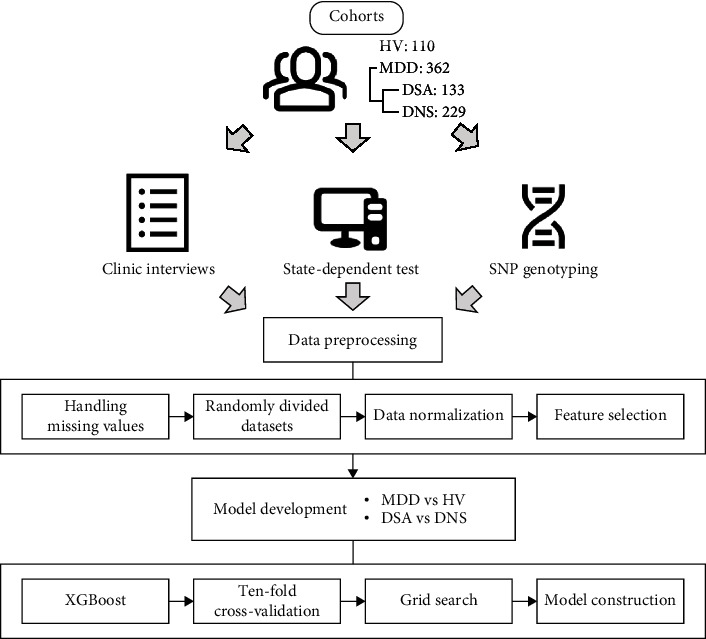
The schema of the study workflow.

Additionally, to understand the importance and contribution of different input characteristics to the models and classification tasks, we added the locally explanatory technique Shapley Additive exPlanations (SHAP), which calculates the relative contribution of each characteristic and explains the ML models [13]. SHAP is commonly employed as a robust approach derived from cooperative game theory, showing its advantageous properties in artificial intelligence interpretation. Moreover, it can display the processing of model decisions. The workflow of this study is illustrated in Figure 2.

The classification abilities of the models were assessed using the area under the receiver operating curve (AUC), sensitivity, specificity, accuracy, positive predictive value, negative predictive value, *F*1-score, and decision curve analysis (DCA). The AUCs of the different models were compared using the DeLong test in MedCalc 19.0.7.

### 2.9. Statistical Analyses

Univariate analyses were performed using the Mann–Whitney *U* test and *t*-test for continuous variables on the basis of whether the data conformed to a normal distribution. Data that conformed to normal distribution are presented by mean ± standard deviation, whereas data that did not conform to normal distribution are expressed as median (quartile range). Categorical variables were analysed using the chi-square test. All the tests were two sided. Statistical significance was set at *p* < 0.05. Statistical analyses were performed using SPSS software (version 25.0; IBM Corp.) and R Studio 4.3.1 (R Foundation for Statistical Computing). We utilized MedCalc software (version 19.0.7) to estimate the required sample size for constructing the classification model. The process and results have been documented in the Supplementary materials.

### 2.10. Role of the Funding Source

The funding sources did not contribute to the study design, data collection, analysis, interpretation, writing of the report, or the decision to submit the paper for publication. All authors had full access to the data in this study and accept responsibility for the decision to submit the manuscript for publication.

## 3. Results

### 3.1. Descriptive Analyses

Descriptive statistics and comparative analyses of HV and patients with MDD, DSA, and DNS are shown in Table 1 and Supplementary Table 1. These analyses were considered descriptive and were not adjusted for other covariates. A total of 362 patients with MDD were included in this study, of whom 133 were diagnosed with DSA. In addition, a control group comprising 110 HV was established. As shown in Table 1, patients with MDD (25 (21–29) years) were younger than HV (26 (22–33) years), and among patients with MDD, those with DSA were younger than those with DNS. There were more males in the HV group (52/110, 47·3%) than in the MDD group (110/362, 30%); however, there was no significant difference between the DSA and DNS groups.

Patients with MDD had more childhood trauma and showed higher impulsivity and aggressiveness traits in all dimensions than did HV (Supplementary Table 1). Among patients with MDD, those with DSA had more childhood trauma in emotional abuse (*p* < 0.001) and physical abuse (*p* < 0.001) and showed higher cognitive impulsivity (*p* = 0.028), physical aggression (*p* = 0.010), anger (*p* = 0.003), and hostility (*p* = 0.032) than did those with DNS.

Patients with MDD showed worse performance in almost all dimensions of the three cognitive function tasks (except for orienting and executive control ability in the ANT and RT in the 2-back task) compared to the other groups. Among the patients with MDD, there were no significant differences between those with DSA and those with DNS. In the 1-back task, those with DSA showed lower ACC than did those with DNS (*p* = 0.001). The specific genotype information about the nine SNPs and the comparison results of the important variables (demographics, predictors, and outcome) between training set and test set are shown in Supplementary materials.

### 3.2. Model Performance

Two sets of ML models were constructed to recognise patients with MDD across the entire population and identify those with DSA among those with MDD. The models that recognised patients with MDD were named D-Models, whereas those that recognised those with DSA were named S-Models.

### 3.3. MDD vs. HV

The dataset was divided into a training set (*n* = 330, 70%) and a validation set (*n* = 142, 30%) using random stratified sampling. There were 253 (76.7%) patients with MDD in the training set and 109 (76.8%) in the validation set. After completing feature selection using the embedded method, D-Models A, B, and C obtained 12, 11, and 12 features, respectively. In the independent validation sets, D-Models A, B, and C that recognised MDD had AUCs of 0.901 (95% CI, 0.850–0.951), 0.938 (95% CI, 0.898–0.977), and 0.928 (95% CI, 0.886–0.969), respectively (Table 2). D-Model B achieved the highest AUC, but there was no statistically significant difference in the AUCs between the three models when compared in pairs (*p* > 0.05). Regarding DCA, if the threshold probability in the clinical decision was in the range of 40–80%, D-Model B provided a greater net benefit than did D-Models A and C. There was no obvious difference in the net benefit between D-Models A and C. According to SHAP analysis, the top five predictors in D-Model B for identifying MDD were ANT-executive control, BIS subscale-cognitive impulsivity, CTQ subscale-emotional neglect, BIS-total score, and SST-negative RT. Furthermore, the DSA from the validation set was chosen to evaluate the recognition capability of D-Model B in this patient cohort. The sensitivity of D-Model B in recognising MDD patients with suicidal tendencies was 97.8% (44/45).

### 3.4. DSA vs. DNS

Patients with MDD were divided into a training set (*n* = 253, 70%) and a validation set (*n* = 109, 30%) using random stratified sampling. There were 93 (36.8%) patients with DSA in the training set and 40 (36.7%) in the validation set. Using the same feature selection method, S-Models A, B, and C obtained 22, 29, and 15 features, respectively. In the independent validation sets, S-Models A, B, and C that recognised DSA had AUCs of 0.702 (95% CI, 0.603–0.802), 0.752 (95% CI, 0.658–0.847), and 0.801 (95% CI, 0.719–0.884), respectively (Table 3). S-Model C achieved the highest AUC value. There was a statistically significant difference between the AUCs of S-Models C and A (*p* = 0.03). Regarding DCA, the overall net benefits of S-Models B and C were better than those of the treat-all models and S-Model A. Additionally, S-Model C performed slightly better than did S-Model B among those with DCA.

S-Model C included 54 features (24, information from clinic interviews; 19, state-dependent test results; and 10, SNP genotyping) in the task of identifying MDD and 58 features (29, information from clinic interviews; 19, state-dependent test results, and 10, SNP genotyping) in the task of identifying DSA among patients with MDD. The embedded method based on the ML algorithm completed the feature selection. This step entails training the XGBoost algorithm on the training set beforehand, acquiring the weight coefficient for each feature, and sequentially eliminating them from largest to smallest to identify the optimal feature set. Finally, after excluding 43 features, 15 features were input into S-Model C, which was considered the most important and optimal feature combination using the XGBoost algorithm. The SHAP analysis revealed that the principal predictors for S-Model C in identifying DSA include total score, duration of the current episode, age of onset, education, and 2-back-RT. In the feature selection process for S-Model C, three specific SNP genotypes were incorporated, namely, rs56834178, rs11288636, and rs68012435. The respective feature importance rankings for these SNP genotypes in S-Model C were 10, 13, and 15.

## 4. Discussion

We used the ML method to establish clinically useful and highly effective predictive models to distinguish patients with MDD from HV and estimate the risk of those with MDD who are likely to attempt suicide. Our results provide clinical psychiatrists with reliable clues and demonstrate the effect of SNP genotyping on DSA.

**Figure 3:**
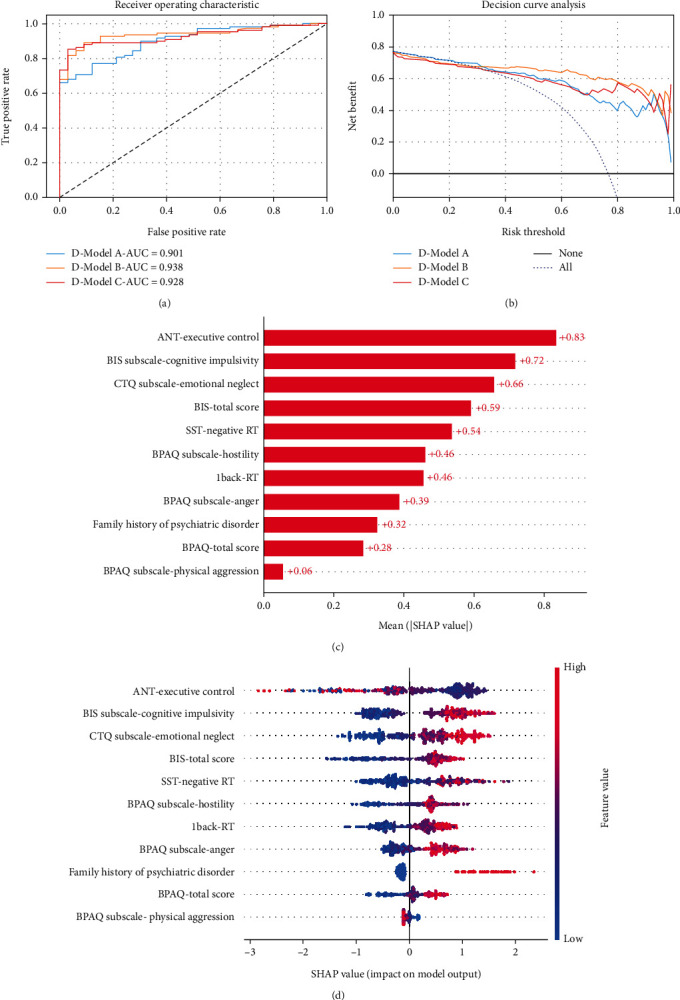
The results from the three ML models and contributions of various features to identify MDD patients. (a) The ROC of three ML models in the validation set. (b) The DCA of three ML models in the validation set. (c) The global bar plot of SHAP values from D-Model B. The features are arranged in descending order based on their contributions from the D-Model B. (d) The beeswarm summary plot of SHAP values from D-Model B. The visualisation indicates the impact of these features on predictions, with colors representing the feature values from high (red) to low (blue). The horizontal position shows whether the feature value leads to a positive or negative prediction. Each point represents a SHAP value of the feature for a specific case.

Our findings suggest that information from clinical interviews or state-dependent tests can distinguish patients with MDD from HV without requiring information directly related to the disease. MDD patients may disguise their depressive symptoms and suicidal intent for stigma, shame, or avoiding more restrictive care [35–37]. Thus, this finding may help for early recognition of depressive symptoms and may save the life of MDD patients. SNP genotyping of *EPHX2* and *P2X2* did not help the model to significantly distinguish patients with MDD. Although previous research has found a correlation between EPHX2 and P2X2 and the severity of depression, some features included in the model may contain similar information. To prevent redundancy in the model, the feature selection stage may have chosen other features with stronger correlations to capture the essential information needed for the classification task. The integration of baseline demographic characteristics, clinical scales (excluding HAMD-24), and data from state-dependent tests allowed D-Model B to exhibit superior classification performance in identifying patients with MDD. Furthermore, D-Model B proved more advantageous for patients compared to the other models. Specifically, at the 50% risk threshold, the net benefit of D-Model B reached 66%, indicating that 66 out of every 100 participants in the study received effective clinical interventions without subjecting the HV to unnecessary interventions. To enhance the interpretability of the model, we added the SHAP method to display the feature importance ranking within the model and the way in which they influenced the model output. The SHAP values associated with D-Model B (refer to Figure 3) highlight that cognitive deficits in executive control, elevated cognitive impulsivity, and significant emotional neglect during childhood were key factors in effectively screening patients with MDD, even in the absence of disease-specific information.

Cognitive decline in patients with MDD has been confirmed in previous studies [38], and the high-impulsivity group exhibited more significant difficulties in resolving conflicts than did the low-impulsivity group when attention switching was involved [39], suggesting the need for special attention in the assessment and intervention of executive control and cognitive impulsivity. Regarding childhood trauma, a meta-analysis found that the prevalences of emotional abuse, emotional neglect, and physical neglect in patients with MDD were high [40] and physical neglect and emotional neglect were the most prevalent types of childhood trauma in Chinese patients with MDD [41]. The present study further demonstrated that emotional neglect is a critical factor in the prediction of MDD.

**Figure 4:**
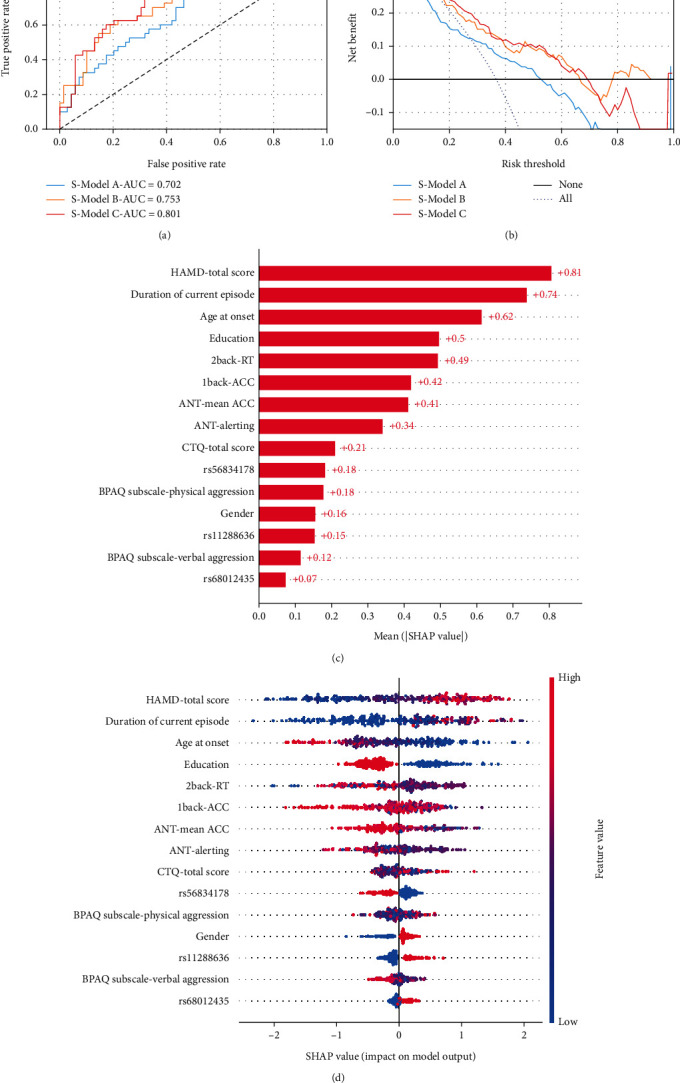
The results from S-Model A, S-Model B, and S-Model C and contributions of various features to identify MDD patients. (a) The ROC of three ML models in the validation set. (b) The DCA of three ML models in the validation set. (c) The global bar plot of SHAP values from S-Model C. (d) The beeswarm summary plot of SHAP values from S-Model C.

When evaluating the risk of suicide attempts, the predictive model with a single dimension could not precisely evaluate the risk of suicide attempts. However, by combining clinical, psychosocial, cognitive, and genetic factors, high performance in classifying suicide attempts in MDD could be achieved. According to the SHAP value of S-Model C (Figure 4), the most important feature for DSA from patients with MDD was the HAMD-24 score, as in previous studies [13, 42]. Patients with MDD with a longer duration of the current episode, younger age at onset, and lower educational level were more likely to attempt suicide than their counterparts. The worst attention (alerting network) and working memory were important cognitive factors in evaluating the risk of suicide. Higher physical aggression, lower verbal aggression, and greater childhood trauma were associated with suicide attempts.

**Figure 5:**
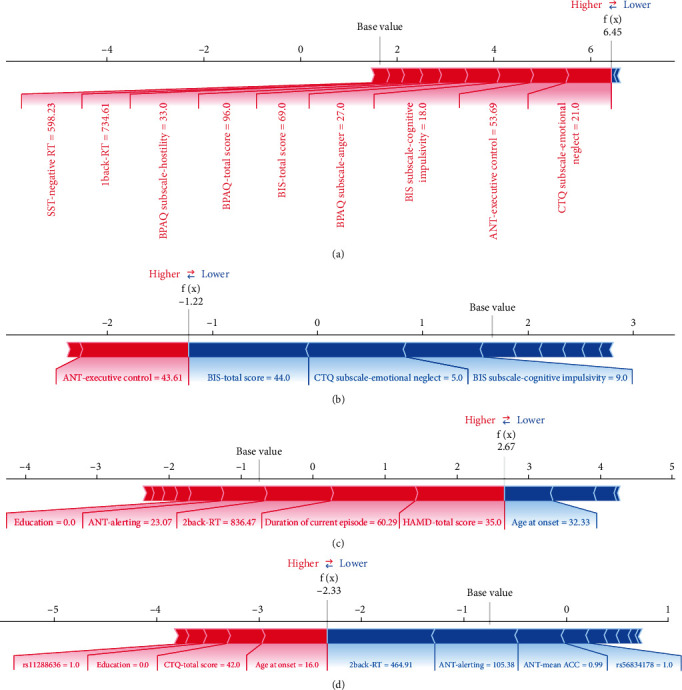
The decision plots of ML models for four randomly selected research objects from the validation set. (a) An MDD case, due to relatively high CTQ subscale-emotional neglect, moderate ANT-executive control, high BIS subscale-cognitive impulsivity, and no other protective factors, D-Model B considered the probability of this patient being MDD to be 99.8%. (b) An HV case, due to relatively low BIS-total score, low CTQ subscale-emotional neglect, and low BIS subscale-cognitive impulsivity, although moderate ANT-executive control accounted for a small portion of the weight, D-Model B considered the probability of this patient being HV to be 77.2%. (c) A DSA case, due to relatively high HAMD-total score, high duration of current episode, and moderate 2-back-RT, although high age at onset accounted for a portion of the weight, S-Model C considered the probability of this patient being DSA to be 93.5%. (d) A DNS case, due to relatively low 2-back-RT, high ANT-alerting, and high ANT-mean ACC, although the role of several risk factors, S-Model C considered the probability of this patient being DNS to be 91.1%. This method could help users better understand the operation and decision-making process of ML models and thus timely intervene in the personalised risk factors of patients.

Three SNPs were selected using the XGBoost algorithm and included in S-Model C. Patients with allele C of rs56834178, allele TTTTTTT of rs11288636, and allele G of rs 68012435 were more likely to attempt suicide than those without. In a previous study, we found an association between several SNP genotypes of *EPHX2* and suicide attempts [23]. However, this study was limited to the analysis of genes and did not consider the combined effects of other factors. ML algorithms are well known for their data processing and feature interactions. This study showed that after adding SNP genotyping to S-Model B, the AUCs of S-Model C increased by 0.049 (*p* > 0.05). After incorporating the information from state-dependent tests and SNP genotyping into S-Model A, the AUCs of S-Model C increased by 0.099 (*p* = 0.03), further illustrating the important role of *EPHX2* in the predictive model of suicide attempts. As the best model in this study, S-Model C could provide a reference for the predictive probability of the risk of suicide attempts in clinical practice, but this is not sufficient. Therefore, we added SHAP, a local interpretation method, to visually demonstrate the important factors affecting and supporting the judgement of the models for each research object, as shown in Figure 5.

In summary, this study successfully established step-by-step predictive ML models first to distinguish patients with MDD from healthy controls and then to estimate the suicide risk of those with MDD. This study also found that SNP genotyping of *EPHX2* can help improve the performance of predictive models for suicide risk. Finally, this study applied decision plots based on SHAP to visualise the personalise risk factors of each patient. Those predicted to be at risk of suicide using the XGBoost model may benefit from interventions. Therefore, our predictive models may be practical and have an additive value.

## 5. Limitations

This study had some limitations. First, this was a single-center study with a limited sample size. Larger samples and further external validation are needed to evaluate the generalisability of the constructed models. Second, cognitive and genetic information was not comprehensive, and more information is required for further research. Finally, neuroimaging data were unavailable for this study.

## 6. Conclusions

Our findings suggest that information from clinical interviews combined with the results of cognitive function tests can distinguish patients with MDD from HV without information directly related to the disease. To evaluate the risk of suicide attempts, comprehensive information was needed to construct a predictive model. In addition, SNP genotyping of *EPHX2* was closely associated with suicide attempts. Our study provides an integrated and clinically applicable model for identifying individuals with MDD and evaluating their risk of suicide attempts.

## Figures and Tables

**Table 1:** Summary of the baseline clinical characteristics comparing HV versus MDD and DNS versus DSA.

	HV (*N* = 110)	MDD (*N* = 362)	*p* value	MDD		*p* value
				DNS (*N* = 229)	DSA (*N* = 133)	
Age (years)	26 (22-33)	25 (21-29)	0.028	25 (22-31)	23 (20-26)	0.025
Gender						
Male	52 (47%)	110 (30%)	0.001	76 (33%)	34 (26%)	0.128
Female	58 (53%)	252 (70%)		153 (67%)	99 (74%)	
Marriage status						
Single	64 (58%)	243 (67%)	0.016	153 (67%)	90 (68%)	0.963
Married	46 (42%)	107 (30%)		69 (30%)	38 (29%)	
divorced or widowed	0(0%)	11(3%)		7(3%)	4(3%)	
Education						
≤12 years	33 (30%)	137 (38%)	0.133	62 (27%)	75 (56%)	<0.001
>12 years	77 (70%)	225 (62%)		167 (73%)	58 (44%)	
Family history of psychiatric disorder						
No	110 (100%)	245 (68%)	<0.001	175 (76%)	70 (53%)	0.857
Yes	0 (0%)	62 (17%)		45 (20%)	17 (13%)	
Family history of major depression disorder						
No	110 (100%)	282 (78%)	0.002	199 (87%)	83 (62%)	0.153
Yes	0 (0%)	25 (7%)		21 (9%)	4 (3%)	
Family history of suicide						
No	110 (100%)	302 (83%)	0.178	217 (95%)	85 (64%)	0.56
Yes	0 (0%)	5 (1%)		3 (1%)	2 (2%)	
History of psychiatric medicine use						
No	110 (100%)	254 (70%)	<0.001	187 (82%)	67 (50%)	0.095
Yes	0 (0%)	53 (15%)		33 (14%)	20 (15%)	
BMI	21.50 (19.71-24.10)	20.06 (18.43-22.55)	<0.001	20.28 (18.42-22.79)	19.77 (18.53-22.43)	0.466
Age at onset (years)	/	/	/	23 (19-28)	19 (16-22)	<0.001
Duration of current episode (weeks)	/	/	/	12 (4-48)	24 (5.75-116.50)	0.006
Recurrent major depressive episode						
No	/	/	/	121 (53%)	42 (32%)	0.27
Yes	/	/	/	98 (43%)	45 (34%)	
HAMD-total score	/	/	/	29.50 (25.75-35)	34 (29-37)	<0.001

HV: healthy volunteers; MDD: major depressive disorder; DNS: depressed non-suicide attempters; DSA: depressed suicide attempters; HAMD: Hamilton MDD Scale-24.

**Table 2:** Classification performance of MDD recognition.

	AUC (95% CI)	Sensitivity (95% CI)	Specificity (95% CI)	Accuracy (95% CI)	PPV (95% CI)	NPV (95% CI)	*F*1-score
D-Model A	0.901 (0.850, 0.951)	91.7% (84.9%, 96.2%)	63.6% (45.1%, 79.6%)	85.2% (78.3%, 96%)	89.3% (82.0%, 94.3%)	70.0% (50.6%, 85.3%)	0.850
D-Model B	0.938 (0.898, 0.977)	93.6% (87.2%, 97.4%)	75.8% (57.7%, 88.9%)	89.4% (83.2%, 94.0%)	92.7% (86.2%, 96.8%)	78.1% (60.0%, 90.7%)	0.894
D-Model C	0.928 (0.886, 0.969)	91.7% (84.9%, 96.2%)	54.5% (36.4%, 71.9%)	83.1% (75.9%, 88.9%)	87.0% (79.4%, 92.5%)	66.7% (46.0%, 83.5%)	0.825

AUC: area under the receiver operating characteristic curve; PPV: positive predictive value; NPV: negative predictive value; D-Model: model for recognising major depressive disorder.

**Table 3:** Classification performance of DSA recognition.

	AUC (95% CI)	Sensitivity (95% CI)	Specificity (95% CI)	Accuracy (95% CI)	PPV (95% CI)	NPV (95% CI)	*F*1-score
S-Model A	0.702 (0.603, 0.802)	52.5% (36.1%, 68.5%)	73.9% (61.9%, 83.7%)	66.1% (56.4%, 74.9%)	53.8% (37.2%, 69.9%)	72.9% (60.9%, 82.8%)	0.660
S-Model B	0.752 (0.658, 0.847)	55.0% (38.5%, 70.7%)	82.6% (71.6%, 90.7%)	72.5% (63.1%, 80.6%)	64.7% (46.5%, 80.3%)	76.0% (64.7%, 85.1%)	0.719
S-Model C	0.801 (0.719, 0.884)	60.0% (43.3%, 75.1%)	81.2% (69.9%, 89.6%)	73.4% (64.1%, 81.4%)	64.9% (47.5%, 79.8%)	77.8% (66.4%, 86.7%)	0.732

AUC: area under the receiver operating characteristic curve; PPV: positive predictive value; NPV: negative predictive value; S-Model: model for recognising depressed suicide attempters.

## Data Availability

We report how we determined our sample size, all data exclusions, all manipulations, and all measures in the study. The data that support the findings of this study are available from the corresponding authors upon reasonable request.

## Additional Points

*Trial Registration*. The trial is registered at ClinicalTrials.gov, registration ID NCT05575713.

## Supplementary Materials

Supplementary Materials See the file Supplementary Materials-0327. The list of supplementary materials: Supplementary Table 1: summary of clinic interviews, state-dependent test, and SNP genotyping information comparing HV versus MDD and DNS versus DSA. Supplementary Table 2: the missing data for the classification tasks of MDD vs. HV. Supplementary Table 3: the missing data for the classification tasks of DSA vs. DNS. Supplementary Table 4: information of SNPs.
