# Supplementary material for: Predictive Models for Suicide Attempts in Major Depressive Disorder and the Contribution of EPHX2: A Pilot Integrative Machine Learning Study
Source: Depress Anxiety. 2024 May 9;2024:5538257. doi: 10.1155/2024/5538257 (PMC11918959; doi:10.1155/2024/5538257)
Supplement: Supplementary Materials — See the file Supplementary Materials-0327. The list of supplementary materials: Supplementary Table 1: summary of clinic interviews, state-dependent test, and SNP genotyping information comparing HV versus MDD and DNS versus DSA. Supplementary Table 2: the missing data for the classification tasks of MDD vs. HV. Supplementary Table 3: the missing data for the classification tasks of DSA vs. DNS. Supplementary Table 4: information of SNPs. [file 5538257.f1.docx]

Supplementary Table 1. Summary of clinic interviews, state-dependent test, SNP genotyping information comparing HV versus MDD and DNS versus DSA.

|  |  | HV （N=110） | MDD （N=362） |  | MDD | |  |
| --- | --- | --- | --- | --- | --- | --- | --- |
|  | |  |  | *p*-value | DNS （N=229） | DSA （N=133） | *p*-value |
| **Information of Clinic Interviews** |  |  |  |  |  |  |  |
| Age(years) | | 26(22-33) | 25(21-29) | 0.028 | 25(22-31) | 23(20-26) | 0.025 |
| Gender | male | 52(47%) | 110(30%) | 0.001 | 76(33%) | 34(26%) | 0.128 |
|  | female | 58(53%) | 252(70%) |  | 153(67%) | 99(74%) |  |
| Marriage status | single | 64(58%) | 243(67%) | 0.016 | 153(67%) | 90(68%) | 0.963 |
|  | married | 46(42%) | 107(30%) |  | 69(30%) | 38(29%) |  |
|  | divorced or widowed | 0(0%) | 11(3%) |  | 7(3%) | 4(3%) |  |
| Education | ≤12 years | 33(30%) | 137(38%) | 0.133 | 62(27%) | 75(56%) | <0.001 |
|  | ＞12years | 77(70%) | 225(62%) |  | 167(73%) | 58(44%) |  |
| Family history of psychiatric disorder | no | 110(100%) | 245(68%) | <0.001 | 175(76%) | 70(53%) | 0.857 |
|  | yes | 0(0%) | 62(17%) |  | 45(20%) | 17(13%) |  |
| Family history of major depression disorder | no | 110(100%) | 282(78%) | 0.002 | 199(87%) | 83(62%) | 0.153 |
|  | yes | 0(0%) | 25(7%) |  | 21(9%) | 4(3%) |  |
| Family history of suicide | no | 110(100%) | 302(83%) | 0.178 | 217(95%) | 85(64%) | 0.56 |
|  | yes | 0(0%) | 5(1%) |  | 3(1%) | 2(2%) |  |
| History of psychiatric medicine use | no | 110(100%) | 254(70%) | <0.001 | 187(82%) | 67(50%) | 0.095 |
|  | yes | 0(0%) | 53(15%) |  | 33(14%) | 20(15%) |  |
| BMI | | 21.50(19.71-24.10) | 20.06(18.43-22.55) | <0.001 | 20.28(18.42-22.79) | 19.77(18.53-22.43) | 0.466 |
| Age at onset (years) |  | / | / | / | 23(19-28) | 19(16-22) | <0.001 |
| Duration of current episode (weeks) |  | / | / | / | 12(4-48) | 24(5.75-116.50) | 0.006 |
| Recurrent major depressive episode | no | / | / | / | 121(53%) | 42(32%) | 0.27 |
|  | yes | / | / | / | 98(43%) | 45(34%) |  |
| HAMD-total score |  | / | / | / | 29.50(25.75-35) | 34(29-37) | <0.001 |
| CTQ-total score | | 33(29-39.25) | 47(37-58.75) | <0.001 | 45.50(36-56) | 53.50(42.75-66.25) | 0.001 |
| CTQ subscale-emotional abuse | | 6(5-8) | 9.5(6-13) | <0.001 | 8.50(6-12) | 12(9-16) | <0.001 |
| CTQ subscale-physical abuse | | 5(5-6) | 5(5-8) | 0.003 | 5(5-7) | 7(5-10) | <0.001 |
| CTQ subscale-sexual abuse | | 5(5-5) | 5(5-6) | 0.019 | 5(5-6) | 5(5-7) | 0.22 |
| CTQ subscale-emotional neglect | | 8(6-11.25) | 15(10-20) | <0.001 | 15(10-19) | 16.50(12.75-22.00) | 0.09 |
| CTQ subscale-physical neglect | | 6(5-9) | 10(7-13) | <0.001 | 10(7-13) | 11(8-14) | 0.056 |
| BIS-total score | | 53.55±7.04 | 65.25±9.50 | <0.001 | 65.23±8.98 | 65.27±10.39 | 0.971 |
| BIS subscale-Cognitive impulsivity | | 12(10-13) | 16(14-18) | <0.001 | 16(13-18) | 17(15-19) | 0.028 |
| BIS subscale-motor impulsivity | | 18(16-20) | 20(17-22) | <0.001 | 19(17-22) | 20(17.75-23) | 0.096 |
| BIS subscale-non-planning impulsivity | | 22(19.75-25) | 28(25-31) | <0.001 | 28(24.75-30) | 28(24.75-32) | 0.141 |
| BPAQ-total score | | 61(52-72.25) | 80(68.25-95) | <0.001 | 78.50(68-93) | 87.50(72.75-101) | 0.003 |
| BPAQ subscale- physical aggression | | 15(12-20) | 20(15-26) | <0.001 | 19(15-25) | 21.50(15.75-28.25) | 0.010 |
| BPAQ subscale-verbal aggression | | 13(10-15) | 14(11-18) | <0.001 | 14(11-18) | 14(11.75-18) | 0.449 |
| BPAQ subscale-anger | | 15(12-18) | 21(17-26) | <0.001 | 20(16-26) | 23(18-27) | 0.003 |
| BPAQ subscale-hostility | | 17(14-21) | 26.5(21-31) | <0.001 | 25.50(20.75-30) | 28(23-33) | 0.032 |
| **Information of State-dependent Test** | |  |  |  |  |  |  |
| ANT-mean-ACC | | 0.99(0.98-1.00) | 0.99(0.97-1.00) | <0.001 | 0.99(0.97-1.00) | 0.98(0.96-1.00) | 0.057 |
| ANT-mean-RT | | 567.71(512.55-630.59) | 596.71(536.33-672.65) | 0.006 | 589.32(536.12-674.66) | 601.05(535.45-663.72) | 0.551 |
| ANT-Alerting | | 17.33(4.71-31.86) | 23.30(6.27-37.00) | 0.032 | 23.16(6.22-37.85) | 23.47(6.18-37.00) | 0.922 |
| ANT-Orienting | | 28.59±26.96 | 24.87±31.21 | 0.267 | 25.25±28.42 | 23.89±37.59 | 0.732 |
| ANT-executive control | | 76.90(11.38-124.53) | 73.74(51.85-98.67) | 0.661 | 74.40(50.81-98.28) | 73.22(54.69-106.40) | 0.475 |
| 1back-ACC | | 0.94(0.88-0.96) | 0.90(0.81-0.95) | <0.001 | 0.90(0.84-0.95) | 0.86(0.72-0.94) | 0.001 |
| 1back-RT | | 653.75(584.14-727.47) | 692.18(619.21-767.23) | 0.001 | 693.98(624.22-774.68) | 689.81(586.50-756.90) | 0.295 |
| 2back-ACC | | 0.74(0.53-0.81) | 0.69(0.52-0.79) | 0.042 | 0.69(0.53-0.79) | 0.67(0.49-0.78) | 0.175 |
| 2back-RT | | 830.15(720.26-918.50) | 835.89(762.75-915.14) | 0.276 | 838.54(770.80-922.90)) | 819.06(748.11-897.47) | 0.282 |
| SST-negative ACC | | 1.00(0.94-1.00) | 0.96(0.96-1.00) | 0.014 | 1.00(0.96-1.00) | 0.958(0.948-1.00) | 0.32 |
| SST-positive ACC | | 1.00(0.94-1.00) | 1.00(0.92-1.00) | <0.001 | 1.00(0.96-1.00) | 0.96(0.92-1.00) | 0.12 |
| SST-neutral ACC | | 1.00(0.96-1.00) | 0.96(0.92-1.00) | 0.003 | 1.00(0.96-1.00) | 0.96(0.92-1.00) | 0.167 |
| SST-suicide ACC | | 1.00(0.96-1.00) | 0.96(0.92-1.00) | <0.001 | 1.00(0.95-1.00) | 0.96(0.92-1.00) | 0.212 |
| SST-negative RT | | 478.23(428.77-554.20) | 542.67(467.69-657.42) | <0.001 | 548.15(465.75-652.13) | 531.12(470.12-669.76) | 0.249 |
| SST-positive RT | | 481.84(428.16-530.89) | 548.93(470.10-640.51) | <0.001 | 552.76(463.40-637.40) | 540.74(484.10-671.44) | 0.277 |
| SST-neutral RT | | 428.25(430.09-546.40) | 545.07(472.41-635.13) | <0.001 | 545.45(469.28-633.54) | 540.85(478.95-653.60) | 0.099 |
| SST-suicide RT | | 483.12(425.59-552.79) | 544.10(469.38-648.30) | <0.001 | 545.38(466.03-649.14) | 543.00(482.65-650.95) | 0.126 |
| P2X2 mRNA expression | | 0.98(0.68-1.36) | 0.92(0.46-1.53) | 0.012 | 0.92(0.46-1.41) | 0.91(0.44-1.57) | 0.292 |
| EPHX2 mRNA expression | | 1.00(0.73-1.36) | 0.96(0.62-1.52) | 0.37 | 0.94(0.62-1.50) | 0.99(0.65-1.69) | 0.626 |
| **Information of SNP Genotyping** | |  |  |  |  |  |  |
| rs11288636 | 0 | 80(73%) | 267(74%) | 0.593 | 178(78%) | 89(67%) | 0.055 |
|  | 1 | 29(26%) | 85(23%) |  | 45(20%) | 40(30%) |  |
|  | 2 | 1(1%) | 8(2%) |  | 4(2%) | 4(3%) |  |
| rs56834178 | 0 | 68(62%) | 214(59%) | 0.191 | 125(55%) | 89(67%) | 0.067 |
|  | 1 | 40(36%) | 127(35%) |  | 88(38%) | 39(29%) |  |
|  | 2 | 1(1%) | 17(5%) |  | 13(6%) | 4(3%) |  |
| rs68012435 | 0 | 80(73%) | 268(74%) | 0.697 | 178(78%) | 90(68%) | 0.082 |
|  | 1 | 28(25%) | 84(23%) |  | 46(20%) | 38(29%) |  |
|  | 2 | 1(1%) | 7(2%) |  | 3(1%) | 4(3%) |  |
| rs7018249 | 0 | 79(72%) | 266(73%) | 0.585 | 177(77%) | 89(67%) | 0.087 |
|  | 1 | 29(26%) | 85(23%) |  | 46(20%) | 39(29%) |  |
|  | 2 | 1(1%) | 8(2%) |  | 4(2%) | 4(3%) |  |
| rs9331942 | 0 | 47(43%) | 146(40%) | 0.918 | 79(34%) | 67(50%) | 0.011 |
|  | 1 | 50(45%) | 170(47%) |  | 118(52%) | 52(39%) |  |
|  | 2 | 12(11%) | 41(11%) |  | 29(13%) | 12(9%) |  |
| rs17466684 | 0 | 76(69%) | 269(74%) | 0.414 | 178(78%) | 91(68%) | 0.163 |
|  | 1 | 31(28%) | 83(23%) |  | 46(20%) | 37(28%) |  |
|  | 2 | 3(3%) | 6(2%) |  | 3(1%) | 3(2%) |  |
| rs9331949 | 0 | 65(59%) | 195(54%) | 0.209 | 114(50%) | 81(61%) | 0.046 |
|  | 1 | 41(37%) | 136(38%) |  | 91(40%) | 45(34%) |  |
|  | 2 | 3(3%) | 26(7%) |  | 21(9%) | 5(4%) |  |
| rs202059124 | 0 | 107(97%) | 320(88%) | 0.008 | 200(87%) | 120(90%) | 0.347 |
|  | 1 | 1(1%) | 28(8%) |  | 20(9%) | 8(6%) |  |
| rs58547167 | 0 | 92(84%) | 317(88%) | 0.555 | 203(89%) | 114(86%) | 0.261 |
|  | 1 | 16(15%) | 41(11%) |  | 23(10%) | 18(14%) |  |
|  | 2 | 0(0%) | 1(0%) |  | 0(0%) | 1(1%) |  |
| rs4149253 | 0 | 77(70%) | 252(70%) | 0.921 | 166(72%) | 86(65%) | 0.086 |
|  | 1 | 30(27%) | 98(27%) |  | 58(25%) | 40(30%) |  |
|  | 2 | 2(2%) | 9(2%) |  | 3(1%) | 6(5%) |  |

Supplementary Table 2. The missing data for the classification tasks of MDD vs HV.

| Feature name | Missing proportion (%) | Feature name | Missing proportion (%) |
| --- | --- | --- | --- |
| Gender | 0.00 | ANT-Orienting | 11.86 |
| Marriage status | 0.21 | ANT-executive control | 11.86 |
| Education | 0.00 | 1back-ACC | 12.29 |
| Family history of psychiatric disorder | 11.65 | 1back-RT | 12.29 |
| Family history of major depression disorder | 11.65 | 2back-ACC | 12.29 |
| Family history of suicide | 11.65 | 2back-RT | 12.29 |
| History of psychiatric medicine use | 11.65 | SST-negative ACC | 4.03 |
| Age | 0.00 | SST-positive ACC | 4.24 |
| BMI | 12.29 | SST-neutral ACC | 4.24 |
| CTQ-total score | 0.42 | SST-suicide ACC | 4.24 |
| CTQ subscale-emotional abuse | 0.42 | SST-negative RT | 4.03 |
| CTQ subscale-physical abuse | 0.42 | SST-positive RT | 4.24 |
| CTQ subscale-sexual abuse | 0.64 | SST-neutral RT | 4.24 |
| CTQ subscale-emotional neglect | 0.42 | SST-suicide RT | 4.24 |
| CTQ subscale-physical neglect | 0.42 | P2X2 mRNA expression | 4.24 |
| BIS-total score | 0.64 | EPHX2 mRNA expression | 3.60 |
| BIS subscale-Cognitive impulsivity | 0.64 | rs11288636 | 0.42 |
| BIS subscale-motor impulsivity | 0.64 | rs202059124 | 3.39 |
| BIS subscale-non-planning impulsivity | 0.64 | rs56834178 | 1.06 |
| BPAQ-total score | 0.85 | rs68012435 | 0.85 |
| BPAQ subscale- physical aggression | 0.85 | rs7018249 | 0.85 |
| BPAQ subscale-verbal aggression | 0.85 | rs9331942 | 1.27 |
| BPAQ subscale-anger | 0.85 | rs17466684 | 0.85 |
| BPAQ subscale-hostility | 0.85 | rs9331949 | 1.27 |
| ANT-mean ACC | 11.86 | rs58547167 | 1.06 |
| ANT-mean RT | 11.86 | rs4149253 | 0.85 |
| ANT-Alerting | 11.86 |  |  |

Supplementary Table 3. The missing data for the classification tasks of DSA vs DNS.

| Feature name | Missing proportion (%) | Feature name | Missing proportion (%) |
| --- | --- | --- | --- |
| Gender | 0 | ANT-mean ACC | 15.47 |
| Marriage status | 0.28 | ANT-mean RT | 15.47 |
| Recurrent major depressive episode | 15.47 | ANT-Alerting | 15.47 |
| Education | 0 | ANT-Orienting | 15.47 |
| Family history of psychiatric disorder | 15.19 | ANT-executive control | 15.47 |
| Family history of major depression disorder | 15.19 | 1back-ACC | 15.75 |
| Family history of suicide | 15.19 | 1back-RT | 15.75 |
| History of psychiatric medicine use | 15.19 | 2back-ACC | 16.02 |
| Duration of current episode | 15.19 | 2back-RT | 16.02 |
| Age | 0 | SST-negative ACC | 5.25 |
| Age at onset | 15.19 | SST-positive ACC | 5.52 |
| Age at current episode | 15.19 | SST-neutral ACC | 5.52 |
| BMI | 15.47 | SST-suicide ACC | 5.52 |
| HAMD-total score | 0 | SST-negative RT | 5.25 |
| CTQ-total score | 0.55 | SST-positive RT | 5.52 |
| CTQ subscale-emotional abuse | 0.55 | SST-neutral RT | 5.52 |
| CTQ subscale-physical abuse | 0.55 | SST-suicide RT | 5.52 |
| CTQ subscale-sexual abuse | 0.83 | P2X2 mRNA expression | 5.25 |
| CTQ subscale-emotional neglect | 0.55 | EPHX2 mRNA expression | 4.70 |
| CTQ subscale-physical neglect | 0.55 | rs11288636 | 0.55 |
| BIS-total score | 0.83 | rs56834178 | 1.10 |
| BIS subscale-Cognitive impulsivity | 0.83 | rs68012435 | 0.83 |
| BIS subscale-motor impulsivity | 0.83 | rs7018249 | 0.83 |
| BIS subscale-non-planning impulsivity | 0.83 | rs9331942 | 1.38 |
| BPAQ-total score | 1.10 | rs17466684 | 1.10 |
| BPAQ subscale- physical aggression | 1.10 | rs9331949 | 1.38 |
| BPAQ subscale-verbal aggression | 1.10 | rs202059124 | 3.87 |
| BPAQ subscale-anger | 1.10 | rs58547167 | 0.83 |
| BPAQ subscale-hostility | 1.10 | rs4149253 | 0.83 |

Supplementary Table 4. Information of SNPs.

| **SNP** | **Gene** | **Chromosome** | **Location** | **Function** | **Genotype** | **Minor Allele** |
| --- | --- | --- | --- | --- | --- | --- |
| rs9331942 | EPHX2 | 8 | 8:27597597 | intron variant | AA | G |
|  |  |  |  |  | AG |  |
|  |  |  |  |  | GG |  |
| rs9331949 | EPHX2 | 8 | 8:27597169 | 3 prime UTR variant | CC | C |
|  |  |  |  |  | CT |  |
|  |  |  |  |  | TT |  |
| rs56834178 | EPHX2 | 8 | 8:27579025 | intron variant | CC | T |
|  |  |  |  |  | CT |  |
|  |  |  |  |  | TT |  |
| rs202059124 | P2X2 | 12 | 12:132620253 | intron variant | CC | T |
|  |  |  |  |  | CT |  |
|  |  |  |  |  | TT |  |
| rs11288636 | EPHX2 | 8 | 8:27489885 | promoter | TTTTTTT.TTTTTTT | TTTTTTT |
|  |  |  |  |  | TTTTTTT.TTTTTTTT |  |
|  |  |  |  |  | TTTTTTTT.TTTTTTTT |  |
| rs17466684 | EPHX2 | 8 | 8:27595330 | intron variant | AA | A |
|  |  |  |  |  | GA |  |
|  |  |  |  |  | GG |  |
| rs68012435 | EPHX2 | 8 | 8:27489871 | promoter | CC | G |
|  |  |  |  |  | GC |  |
|  |  |  |  |  | GG |  |
| rs7018249 | EPHX2 | 8 | 8:27514672 | intron variant | AA | A |
|  |  |  |  |  | GA |  |
|  |  |  |  |  | GG |  |
| rs7829267 | EPHX2 | 8 | 8:27535469 | intron variant | CC | T |
|  |  |  |  |  | CT |  |
|  |  |  |  |  | TT |  |
| rs4149253 | EPHX2 | 8 | 8:27538691 | Exon-synonymous | AA | A |
|  |  |  |  |  | AG |  |
|  |  |  |  |  | GG |  |
